# The complete mitochondrial DNA sequence and the phylogenetic position of *Rhabdophis tigrinus* (Reptilia: Squamata)

**DOI:** 10.1080/23802359.2016.1155092

**Published:** 2016-03-28

**Authors:** Dan Zhao, Huan Liu, Wen-Ge Zhao, Peng Liu

**Affiliations:** College of Life Science and Technology, Harbin Normal University, Harbin, P.R. China

**Keywords:** Colubridae, mitogenome, phylogenetic tree, *Rhabdophis tigrinus*

## Abstract

The complete mitochondrial genome of *Rhabdophis tigrinus* (Reptilia: Squamata) is presented for the first time in this study. It is a circular molecule of 17 415 bp in length (GenBank accession no. KU641019), consisting of 13 protein-coding genes, 22 transfer RNA genes, two ribosomal RNA genes (12S and 16S rRNA) and two control regions (D-loop), with the typical gene order and direction of transcription in Serpentes. The overall base composition is 33.65% A, 26.70% C, 13.16% G and 26.49% T. Mitochondrial genomes analyses based on NJ method yield phylogenetic trees, including 14 reported snakes belonging to four families (Colubridae, Elapidae, Viperidae and Typhlopidae). These molecular data presented here provide a useful tool for systematic analyses of genus *Rhabdophis* and family Colubridae.

Chinese Tiger Snake (*Rhabdophis tigrinus*) is a medium-sized snake, belonging to the genus *Rhabdophis* of family Colubridae. This snake is widely distributed in the southeast of Russia, Korean Peninsula, Japan and mainland of China (Zhu et al. 2014). At present, *R. tigrinus* is mainly divided into three subspecies, but it is difficult to distinguish based on the morphology traits (Zhao & Adler 1993; Zhao 2006). Now mitochondrial genome (mtDNA) is widely used in phylogenetic and evolutionary studies (Crimi & Rigolio 2008). It is the first time to sequence the complete mitochondrial genome of *R. tigrinus* mainly based on the muscle sample using primers for the walking strategy and accurate PCR. The specimen was collected from Jilin Province of China (42°35′39.7″N, 127°50′41.9″E) and was stored in Zoological and Botanical Specimen Museum of Harbin Normal University (its accession no. is HRB150458).

In this study, the complete mtDNA of *R. tigrinus* is 17 415 bp long, consisting of 13 protein-coding genes, 22 transfer RNA genes, two ribosomal RNA genes (12S and 16S rRNA) and two control regions (D-loop). The nucleotide composition was 33.65% A, 26.70% C, 13.16% G and 26.49% T. Accurate annotation of the mitochondrial genome sequence was submitted to GenBank (accession no. KU641019).

Within the mitochondrial genome of *R. tigrinus*, there are 12 reading frame overlaps (share 1–10 nucleotides) and four intergenic spacers (range from 1 to 22 bp). Besides *ND6*, D-loop and nine tRNA genes are on the L-strand, other genes are encoded in the H-strand. In 13 protein-coding genes, except *ND1*, *COI* and *ND6* with ATA, *ND2* and *ND3* with ATT, *ND4L* with GTG, *ND5* with ATC, the other six genes begin with ATG as start codon. *ATP8*, *ATP6*, *ND4L*, *ND4* and *ND5* genes are terminated with TAA as stop codon, *ND1*, *COII COIII*, *CYTB* and *ND3* end with a single stop nucleotide T, *ND2* ends with TAG, *ND6* ends with AGA and *COI* ends with AGG. The 22 tRNA genes with the size ranging from 57 to 73 bp are interspersed along the whole genome. The sequence length of the 12S and 16S rRNA is 925 and 1461 bp, two D-loop regions are 1072 and 1225 bp. In the WANCY cluster of tRNA genes, a 38 bp sequence is considered as the putative L-strand replication origin (OL).

Mitochondrial genomes analyses based on MP, ML and NJ produced the same phylogenetic trees, including 14 reported snakes belonging to four families (Colubridae, Elapidae, Viperidae and Typhlopidae) (Figure 1). It appeared that genus *Lycodon* and *Elaphe* formed a monophyletic group except genus *Rhabdophis*, while they belong to the same family Colubridae. It helps to study the systems of the related species of genus *Rhabdophis* and the genetic structure of family Colubridae (Kumazawa et al. 1996; He et al. 2010; Yan et al. 2014; Oh et al. 2015; Qian et al. 2016; Wan et al. 2016).

**Figure 1.**
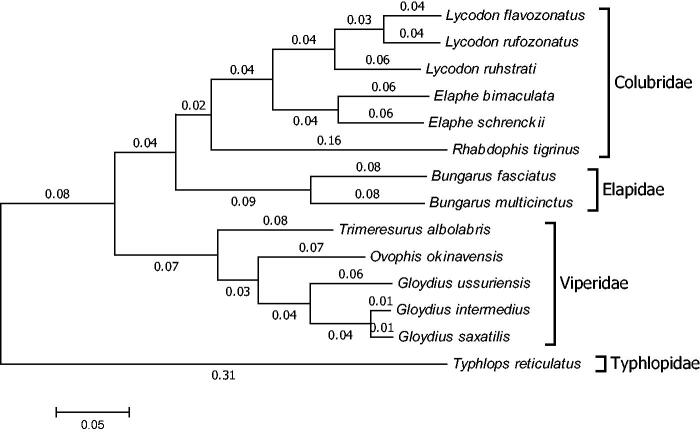
Phylogenetic tree generated using the neighbour-joining method based on complete mitochondrial genomes of some species of Serpentes. *Lycodon flavozonatus* (NC_028730), *Lycodon rufozonatus* (NC_024559), *Lycodon ruhstrati* (KJ179951), *Elaphe bimaculata* (KM065513), *Elaphe schrenckii* (NC_027605), *Rhabdophis tigrinus* (KU641019), *Bungarus fasciatus* (EU579523), *Bungarus multicinctus* (NC_011392), *Trimeresurus albolabris* (NC_022820), *Ovophis okinavensis* (NC_007397), *Gloydius ussuriensis* (NC_026553), *Gloydius intermedius* (NC_025560), *Gloydius saxatilis* (KM434236) and *Typhlops reticulatus* (NC_010971).
